# LASIK Videos on TikTok: A Content Analysis of the Top 100 Videos

**DOI:** 10.1155/2024/8810500

**Published:** 2024-05-21

**Authors:** Firas F. Haddad, Joanna S. Saade

**Affiliations:** ^1^American University of Beirut, Faculty of Medicine, Beirut, Lebanon; ^2^Department of Ophthalmology, American University of Beirut Medical Center, Beirut, Lebanon

## Abstract

**Introduction:**

Social media has increasingly become a prominent source of health information. Platforms like TikTok that allow for videos to reach millions of viewers have become among the most common platforms to share and receive health information. Laser in situ keratomileusis (LASIK) videos and patients' experiences are commonly discussed on social media. The quality of these videos remains to be assessed. The aim of this study is to evaluate the content, quality, and reach of the top 100 videos related to LASIK eye surgery on TikTok.

**Methods:**

Video quality was assessed using the DISCERN, JAMA, and GQS instruments which have all been proven to be both reliable and valid.

**Results:**

100 videos were included in the study. Results showed that the videos have an immense reach with a total view count of 245 million views and 21.9 million likes. Two thirds of the videos were posted by personal accounts as compared ophthalmologists that only constituted 26% of the content. Healthcare professionals produced higher quality videos compared to nonhealthcare professionals (*p* < 0.0001) although there was no significant difference in video duration (*p*=0.18). Increased duration, view count, comments, shares, saves, and views/day were all associated with increased DISCERN score and quality of the videos. Educational videos were of higher quality compared to entertainment videos, and videos outlining the procedure details had the highest quality score.

**Conclusions:**

LASIK videos on TikTok have established a wide reach, whereby viewers are highly interacting and viewing these videos. It appeared that viewers interacted more with the higher quality videos. Ophthalmologists approximately only contributed to a quarter of the videos analyzed in this study. This highlights the need for ophthalmologists to establish a presence on TikTok and produce high quality videos.

## 1. Introduction

Social media is exponentially growing to integrate itself in the field of healthcare. Its efficiency and its ease of access in receiving information at the click of a few buttons have made it the first resort for patients hoping to acquire information on medical conditions and procedures [1]. However, the lack of regulation in social media platforms has allowed the spread of misinformation, which may be detrimental to the public. In recent years, healthcare workers have gradually resorted to social media to spread awareness, educate the general public, and debunk false information and myths spread online [2].

As the world's fastest growing online platform, TikTok has around 1 billion active users per month [3, 4]. Although it does not rank in the top three social media platforms, this can be due to the fact that it is among the youngest, established only in 2016. It initially attracted a younger demographic; however, it is now extending to all age groups and ranks among the fastest growing social media platforms [5]. It has a personalized feature referred to as the “For You Page,” which includes videos especially tailored for the viewer's likes and interests. Through a simple interface, viewers can access hundreds and thousands of videos related to any topic of interest. Like other social media platforms, it has been used with the intent of spreading information, particularly health-related information. However, videos on TikTok are most commonly shared in 15 second, 1 minute, and 3 minute timeframes with exceptional videos extending beyond that. This sets a limitation on the amount of adequate knowledge that can be shared and explained in such a short time compared to other social media platforms. These limitations have not stopped patients from resorting to social media for health information. A study found that 75–80% acquired health information online [6]. This emphasizes the need to assess the quality of the information shared on this platform to avoid the spread of misinformation and ensure proper education of the general public.

Laser in situ keratomileusis (LASIK) correction of refractive errors is among the most common procedures conducted by ophthalmologists worldwide [7]. There are many misconceptions about LASIK spread on social media [8]. Many of these misleading videos are shared by patient's individual experiences and are not evidence based. As such, it is essential to assess the quality of videos being published about LASIK, the type of content being produced, the characteristics of their content creator, and most importantly, the reach of these videos.

The aim of this study is to analyze the top 100 videos related to LASIK on TikTok and to study the type of video, its creator's credentials, the content, the quality, and the reach.

## 2. Methods

### 2.1. Video Selection

**Figure 1:**
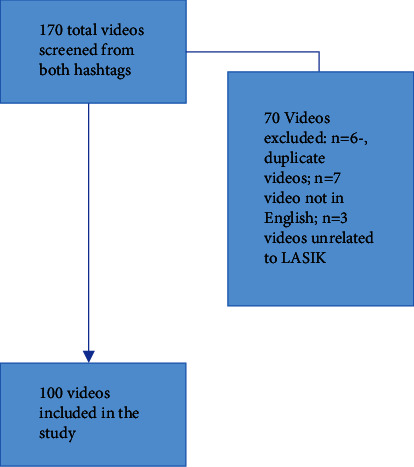
Video selection and the inclusion flowchart.

The two most popular hashtags #lasik (622.1M views) and #lasiksurgery (62.0M) were used to extract the videos.  Figure 1 outlines the video selection process. Videos were downloaded on May 1^st^, 2023. All data extraction was done based on the downloaded videos to prevent new videos from emerging in the hashtag.

### 2.2. Data Extraction

The general information extracted included the duration of the video and the date the video was published on TikTok. The number of days the video has been on TikTok was calculated by subtracting May 1^st^, 2023 from the date the video was published online. The views/day was calculated by dividing the total number of views of the video by the number of days the video has been available on TikTok.

Reach of the video was assessed via the total number of views, likes, comments, shares, and number of saves of the video. The content creator was evaluated by extracting the number of account followers, TikTok certification, and the content creator category (ophthalmologist, nonophthalmology doctor, optometrist, nurse, resident/student, personal account, brand, or organization). In regards to the type of video, videos were considered either procedure details, comedy, patient experience, Question and Answer (Q&A), case discussion, or negative patient experience. The purpose of the video was categorized as either educational or for entertainment purposes. Then, a yes/no input was extracted for whether the video mentioned LASIK surgery details, specific LASIK techniques, visual outcomes and satisfaction, use of visual aids, or risks of LASIK.

### 2.3. Video Quality

Video quality was assessed by using three international valid and reliable scoring systems for health information on the internet. The first, the DISCERN scoring instrument, consists of 15 questions each graded between 1 and 5. A total score is then divided by 15 to get an average score between 1 and 5, reflecting the quality of the video. The instrument looks at the video's relevance and currency, its bias and subjectivity, whether it discusses the range of treatment choices and their risks/benefits, and whether it discusses decision-making and future projections of the treatment choice [9]. The second tool used was the JAMA score. It consists of 4 items each receiving a score of either 0 or 1 to calculate a total score out of 4. The score looks at the following 4 criteria: authorship, attribution, disclosures, and currency [10]. The third score was the GQS (Global Quality Scale). It consists of a scale of 5 points, with increasing points indicating higher quality, better flow, and more relevant information presented with little bias [10]. Higher scores in all three scales reflect a higher quality of video, and all three scoring systems have been used in the literature to quantify the quality of health information videos.

### 2.4. Data Analysis

Data analysis was conducted on Microsoft Excel and SPSS. The means, standard deviations, median, and interquartile ranges of the different reach parameters were calculated on Excel. Correlation coefficients and their *p* values were calculated on SPSS. The Kruskall–Wallis test was used to test the difference in mean DISCERN scores against different parameters.

## 3. Results

One-hundred videos were included in the study with a total of 245,173,500 views, 21,911,733 likes, 214,608 comments, 748,326 shares, and 1,036,173 saves.  Table 1 outlines the average of different data parameters of the videos included.

**Figure 2:**
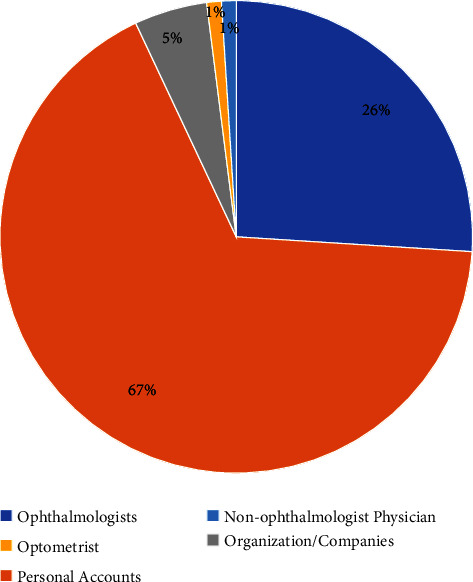
Video distribution by the creator type.

Forty-two videos (42.0%) were posted by small accounts with a follower count <50,000; 21 videos (21%) were posted by medium sized accounts (50,000–200,000); and 37 videos (37%) were posted by large accounts (>200,000). Seventeen percent of videos (*n* = 17) were posted by accounts that are TikTok certified, while the remaining did not have such verification.  Figure 2 outlines the distribution of videos among the different content creator types.

**Figure 3:**
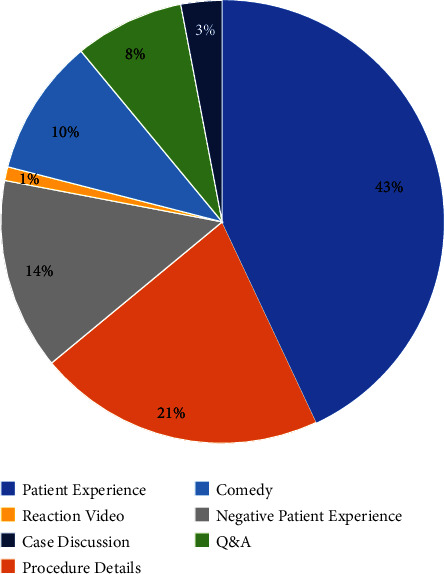
Video distribution by the video type.

Seventy-four percent were educational videos while 26% were entertainment videos. Twenty-eight percent mentioned LASIK procedure steps, 25% mentioned visual outcomes and satisfaction, 15% mentioned specific LASIK techniques, 15% mentioned the risks and complications associated with LASIK, and 8% used visual aids in the videos.  Figure 3 outlines the video distribution among the different video types.

Table 2 shows the correlation between the 3 scoring systems and different parameters related to the reach of the videos.

The *r* correlation coefficient presented in Table 2 range between 0.10 and 0.30, indicating a modest positive correlation, and some of the parameters have significant correlation coefficients while others did not. Increased duration, view count, comments, shares, saves, and views/day were associated with increased DISCERN score and quality of the videos. Increased view count, shares, and saves were associated with the increased JAMA score. Increased duration, view count, comments, shares, and saves were associated with the increased GQS score. This indicates that the longer the video is, the better quality the video was rated in both the DISCERN and GQS scores. The average video length produced by healthcare professionals (ophthalmologist, nonophthalmologist doctor, and optometrists) was 37.18 ± 18.44 seconds whereas the average video duration produced by nonhealthcare professionals was 48.47 ± 42.14 seconds (*p* = 0.18). Higher quality videos were viewed, commented on, shared, and saved more than their lower quality counterpart, which indicates that viewers were enjoying higher quality videos rather than the lower quality ones.  Table 3 presents the DISCERN score of the videos grouped by account size, creator type, type of video, and purpose of video.

## 4. Discussion

Social media has increasingly become a source of health information for the public due to its low cost, efficiency, and simplicity [1]. TikTok is among the most prominent platforms to share and receive health-related knowledge due to the high reach these videos get compared to other platforms [2]. Our study highlights a significant reach for LASIK-related videos on the platform with a total view around 250 million views and an average of 2.5 million views/video. Our study also highlights that TikTok videos about LASIK are still of poor quality. With an average DISCERN score of 1.67/5, the videos are still poor in quality and have major shortcomings. With that in mind, ophthalmologists should ride the wave and resort to TikTok to spread educational videos related to LASIK eye surgery. In our cohort, only 26% of videos were posted by ophthalmologists.

In addition, healthcare professionals created the videos with the highest quality, which further emphasizes the need for doctors in general to increase their presence on the platform due to their ability to create unbiased and high quality content. Nonhealthcare professionals created the highest number of the videos with the least quality score, which has been a trend found in other studies as well. Healthcare professionals and nonhealthcare professionals produced videos nonsignificant differences in duration; however, healthcare professionals' videos were of higher quality.

The duration, number of views, shares, saves, comments, and views/day all had a moderate correlation with DISCERN quality. This indicates that viewers were interacting with high quality videos more than their lower quality counterparts. This finding has been reported in other studies as well [11]. The duration, number of views, shares, saves, and comments were all moderately correlated with GQS quality. The JAMA score did not highlight the same association. It may be due to the limited and simpler nature of the scoring system. The JAMA score consists of 4 yes/no questions as compared to 15 scaled questions in the DISCERN scoring instrument. In addition, the JAMA tool addresses more specific questions pertaining to the authorship, attribution, disclosure, and currency of the video, whereas the GQS is more of a broad scaling from 1 to 5 of the quality of the video.

Medium-sized accounts had the tendency to produce the highest quality videos. Videos outlining procedure details had the highest quality, possibly because these videos were exclusively published by healthcare professionals who are the most knowledgeable in the field. Comedy and negative patient experiences had the lowest quality. Comedy videos produced for entertainment purposes were of low quality in the present study; an expected finding since the purpose of the video is for entertainment rather than to educate the general public. It would be interesting for future studies to assess whether comedy videos that are educational and contain informative content perform superiorly to both comedy videos for entrainment purposes or educational material lacking comedy. Negative patient experience videos were created mostly by personal accounts, resulting in their lower quality. As such, there is a need for ophthalmologists to address the risks of LASIK eye surgery in a nonbiased manner to prevent the spread of misinformation created by low quality videos of negative patient experiences.

Different studies assessing the use of TikTok in their respective fields have reached results similar to ours [12, 13]. A study by Siegal et al. on TikTok videos about varicoceles found that healthcare professionals published significantly better videos than their nonhealthcare counterparts [14]. A study by Dubin et al. on men's health information on TikTok established the huge reach health information videos have on TikTok and that the video quality was poor and published mostly by nonhealthcare professionals [15]. A study by Chen et al. found a similar correlation between the duration of the video and the video quality (DISCERN and GQS), confirming that high quality videos tended to be of longer duration, as reported in this present study [16].

The assessment of the misinformation on social media has been studied before. A study published in 2021 found that the most common topics with misinformation on social media were vaccines, drugs/smoking, noncommunicable diseases, medical treatment, and pandemics. The study showed that 30% of the included posts related to medical treatments contained misinformation. This number can rise up to 87% in posts related to smoking and drug use [17]. Another study published in 2022 assessed a similar topic. The study included no studies related to misinformation on TikTok, which emphasizes the need for further studies to assess the quality of health information on this platform. The study reported a very sharp increase in publication and citation count on papers related to misinformation on social media, highlighting the acknowledgement of the influence of social media platforms on awareness and health information [18].

Our study is not without its limitations. First, the study is a cross-sectional study and thus causal relationship cannot be established. Second, only videos done in the English language were included which limits out ability to generalize our results worldwide. Lastly, the sample size was limited to 100 videos. Other studies are encouraged to analyze videos with a greater sample size.

## 5. Conclusion

Our study highlights that although TikTok videos regarding LASIK have a very wide reach, they are mostly of poor quality. With better knowledge, ophthalmologists are urged to produce more videos since they only constitute 26% of the top videos. The results show that viewers were interacting and viewing better quality content. One prominent issue to address is the negative patient experiences. Such videos were numerous and were of very poor quality. As such, ophthalmologists should counter the effects of these videos by objectively discussing the risks and complications of LASIK eye surgery.

## Figures and Tables

**Table 1:** Average, standard deviation, range, and median (IQR) of parameters related to the reach of the videos.

	Average ± St. deviation	Range	Median (IQR)
Duration (s)	45.31 ± 37.31	5–179	39 (38)
Views	2,451,735 ± 4,261,401.8	47000−31,700,000	1100000 (1931625)
Likes	219,117.33 ± 385,734.43	7852−2,300,000	80600 (183050)
Comments	2146.08 ± 3607.63	72−23,900	719.5 (1737.75)
Shares	7483.27 ± 21,793.13	3–161,100	1404 (4539)
Saves	10,361.73 ± 22,271.79	61–162,100	3214 (5945.25)
Views/Day	9360 ± 26,520.87	176.75–212,751.68	1933.12 (5008.20)
DISCERN	1.67 ± 0.47	1–2.73	1.5 (0.67)
JAMA	1.96 ± 0.67	1–3	2 (0)
GQS	1.76 ± 0.78	1–4	2 (1)

**Table 2:** Correlation between the three scoring systems and the reach of the videos.

	DISCERN (*p* value)	JAMA (*p* value)	GQS (*p* value)
Duration	0.21 (0.03)	0.16 (0.11)	0.29 (0.003)
Views	0.24 (0.018)	0.20 (0.049)	0.20 (0.049)
Likes	0.10 (0.30)	0.11 (0.25)	0.14 (0.18)
Comments	0.27 (0.0072)	0.16 (0.10)	0.23 (0.02)
Shares	0.29 (0.0034)	0.22 (0.029)	0.27 (0.007)
Saves	0.26 (0.01)	0.27 (0.005)	0.30 (0.002)
Views/Day	0.20 (0.04)	0.15 (0.12)	0.17 (0.089)

**Table 3:** DISCERN score by account size, creator type, type of video, and purpose of video.

	Mean DISCERN Score	*p* value	Median (IQR)
Account size		*p* < 0.00001	
Small	1.44		1.34 (0.3)
Medium	2.15		2.33 (0.75)
Large	1.64		1.5 (0.55)
Creator type		*p* < 0.0001	
Healthcare professional (ophthalmologists, nonophthalmologist doctor, and optometrist)	2.17		2.33 (0.40)
Nonhealthcare professionals	1.45		1.4 (0.3)
Type of video		*p* < 0.0001	
Comedy	1.08		1 (0.2)
Negative patient experience	1.37		1.33 (0.2)
Patient experience	1.59		1.5 (0.3)
Procedure details	2.15		2.33 (0.64)
Q&A	1.92		1.92 (0.93)
Purpose		*p* < 0.0001	
Education	1.77		1.6 (0.87)
Entertainment	1.35		1.32 (0.5)

## Data Availability

The data used to support the findings of this study are available on request from the corresponding author.

## Additional Points

*Synopsis*. This study shows that TikTok videos discussing LASIK surgery are of poor quality and lack essential information. The study also shows that healthcare professionals have not established a presence on the platform.
